# Antioxidant Dietary Fiber Sourced from Agroindustrial Byproducts and Its Applications

**DOI:** 10.3390/foods12010159

**Published:** 2022-12-28

**Authors:** Jorge E. Angulo-López, Adriana C. Flores-Gallegos, Juan A. Ascacio-Valdes, Juan C. Contreras Esquivel, Cristian Torres-León, Xochitl Rúelas-Chácon, Cristóbal N. Aguilar

**Affiliations:** 1 Food Research Department, School of Chemistry, Universidad Autónoma de Coahuila, Saltillo 25280, Coahuila, Mexico; 2 Research Center and Ethnobiological Garden, Universidad Autónoma de Coahuila, Viesca 27480, Coahuila, Mexico; 3 Department of Food Science and Technology, Autonomous Agrarian University Antonio Narro, Calzada Antonio Narro 1923, Saltillo 25315, Coahuila, Mexico

**Keywords:** dietary fiber, byproducts, functional ingredient, antioxidant, bioactive compounds

## Abstract

Agroindustrial activities generate various residues or byproducts which are inefficiently utilized, impacting the environment and increasing production costs. These byproducts contain significant amounts of bioactive compounds, including dietary fiber with associated phenolic compounds, known as antioxidant dietary fiber (ADF). Phenolic compounds are related to the prevention of diseases related to oxidative stress, such as neurodegenerative and cardiovascular diseases. The mechanism of ADF depends on its chemical structure and the interactions between the dietary fiber and associated phenolic compounds. This work describes ADF, the main byproducts considered sources of ADF, its mechanisms of action, and its potential use in the formulation of foods destined for human consumption. ADF responds to the demand for low-cost, functional ingredients with great health benefits. A higher intake of antioxidant dietary fiber contributes to reducing the risk of diseases such as type II diabetes, colon cancer, obesity, and kidney stones, and has bile-acid retention–excretion, gastrointestinal laxative, hypoglycemic, hypocholesterolemic, prebiotic, and cardioprotective effects. ADF is a functional, sustainable, and profitable ingredient with different applications in agroindustry; its use can improve the technofunctional and nutritional properties of food, helping to close the cycle following the premise of the circular economy.

## 1. Introduction

The fruit and vegetable processing industries generate large amounts of waste [1], which can have different uses in the food industry [2] as functional ingredients [3]. These byproducts are an abundant and economical source of valuable compounds such as polyphenols, vitamins, carotenoids, and dietary fiber [1,3,4]. Dietary fiber is a component of plant cell walls, responsible for providing structural support to fruits and vegetables. Chemically, DF is constituted of various carbohydrate polymers, including homopolysaccharides, heteropolysaccharides, lignans, oligosaccharides, resistant starches, gums, and mucilages [5]. Some byproducts of tropical fruit processing identified as potential sources of antioxidant dietary fiber are mango (*Mangifera indica* L.), avocado (*Persea americana* Mill.), pineapple (*Ananas comosus* L.), papaya (*Carica papaya* L.), guava (*Psidium guajava* L.), grape pomace [6], and carrot [7], among others. Some of these byproducts even have higher concentrations of dietary fiber and polyphenols than some cereals [4,8]. These fibers are characterized by the fact that endogenous enzymes do not hydrolyze them in the human small intestine and their consumption is associated with beneficial health effects, including regulation of intestinal transit and prevention or treatment of cancer, diabetes, and cardiovascular disease [3,9]. DF can regulate glucose and cholesterol levels [3].

Research has shown that fruit and vegetable processing byproducts such as peels, seeds, and pomace are excellent sources of antioxidant dietary fiber, with nutritional and physicochemical properties that are important in the food industry [10]. In accordance with the above, the general objective of this review article is to describe the most important aspects of antioxidant dietary fiber, the main byproducts considered to be sources of ADF, and ADF’s mechanisms of action and use as a functional ingredient in foods.

## 2. Antioxidant Dietary Fiber (ADF)

Antioxidant dietary fiber can be defined as a dietary fiber concentrate that contains significant amounts of natural antioxidants associated with the fiber matrix. These antioxidants are mainly phenolic compounds [11,12]. This term was first introduced to describe a natural product found in grape pomace that is rich in dietary fiber and antioxidants [12]. For a material to be considered an antioxidant dietary fiber it must meet the following conditions: (i) The dietary fiber content must be greater than 50% on a dry basis, measured via the AOAC method (AOAC enzymatic–gravimetric method); (ii) 1 g of the antioxidant dietary fiber must have the capacity to inhibit lipid oxidation equivalent to at least 200 mg of vitamin E (measured via the thiocyanate procedure) and a free-radical-scavenging capacity equivalent to at least 50 mg of vitamin E (measured via the DPPH method); (iii) the antioxidant capacity must be specific to the material and must not be due to other compounds added or released because of previous enzymatic chemical treatments [3,12].

## 3. Phenolic Compounds Associated with Dietary Fiber

Antioxidant dietary fiber is characterized by having associated bioactive compounds [4,12] that are mainly polymeric polyphenols and low-molecular-weight polyphenols [13]. Phenolic compounds are secondary metabolites of plant origin. They are part of the plant’s protection mechanisms against environmental factors and diseases. Their presence contributes to the functional properties and influences the color, smell, and flavor of multiple plants, vegetables, and fruits [3]. These are bound to the dietary fiber of the cell wall [4,12] through hydrophobic aromatic rings and hydrophilic hydroxyl groups [8]. This association between dietary fiber and phenolic compounds through ionic, covalent, or hydrogen bonds is key to the recognition of potential antioxidant dietary fibers. This makes it possible to associate the benefits of antioxidants with the properties of fiber [6,12,14]. The interaction between polyphenols and dietary fiber regulates the release of bioactive compounds from their matrices and their absorption in the gastrointestinal tract. Additionally, these interactions depend on the type of antioxidant and its structure, size, and number of functional groups [8]. Depending on their structure, antioxidants can have one or more phenolic groups. More than 8000 phenolic compounds have been described. They are mainly classified into flavonoids and nonflavonoids [5]. Environmental factors such as pH, temperature, and ionic strength can influence the polyphenol content in plant cell walls [8].

These polyphenols can be released during digestion (making them available for absorption) or released in the colon after fiber fermentation, where they can contribute to benefit intestinal health or be excreted in the feces [8]. Phenolic content can be used as an important indicator of antioxidant capacity and can be used as a preliminary screen for any product when it is intended to be used as a natural source of antioxidants in functional foods [15,16]. Some studies have reported a positive correlation between the total phenolic content and antioxidant capacity of fruit extracts. According to Martinez et al. [16], exotic fruit fibers can be considered good sources of natural compounds with significant antioxidant activity. Table 1 presents the contents of total dietary fiber and phenolic compounds in some of the most important byproducts considered sources of ADF.

Antioxidant dietary fiber is characterized by higher levels of TPC compared to aqueous extracts of other food byproducts; examples include the residues of juice production (apple, 46 mg GAE/g; pear, 13 mg GAE/g; and red beet, 92 mg GAE/g); waste from the canning industry (artichoke, 43 mg GAE/g; asparagus, 89 mg GAE/g; and tomato, 12 mg GAE/g); crop residues (broccoli, 30 mg GAE/g; cucumber, 18 mg GAE/g; escarole, 34 mg GAE/g; and chicory, 14 mg GAE/g); and minor crops (goldenrod, 112 mg GAE/g and hay, 63 mg GAE/g) [16,32].

Table 2 shows the antioxidant activities reported for byproducts and dietary fiber concentrates. The types of phenolic compounds and their concentrations in the fruit and its byproducts depend on several factors: variety, maturity stage, and harvest season; environmental factors (soil and climate); and extraction method and type of solvent used [16,22].

## 4. Byproducts as Sources of Antioxidant Dietary Fiber

Traditionally, byproducts from the milling of cereals such as wheat, corn, sorghum, and other cereals have been used as sources of fiber [33]. However, dietary fiber from fruit and vegetable byproducts has the advantage of containing a more significant portion of soluble dietary fiber (33%) compared to that found in cereals (7%). According to dietary fiber requirements, it is essential that a fiber source has a balance between soluble and insoluble fiber fractions, i.e., the water-soluble fraction should represent between 30 and 50% of the total dietary fiber [34].

Recent research has focused on obtaining antioxidant dietary fiber from plant food byproducts [11]. Agroindustrial byproducts are of interest since they are inexpensive and available in large quantities [35]. These byproducts are sources of dietary fiber and other bioactive compounds, including vitamins and phenolic compounds [4]. Dietary fiber obtained from fruit and vegetable byproducts (peels, seeds, and pomace) has excellent physicochemical properties. This type of fiber plays an important role in the food and pharmaceutical industries, so this type of fiber extraction has an assured market potential [36].

The processing of ADF can affect the content of fiber and bioactive compounds. High temperatures can cause partial degradation of some components of soluble fiber [35,37]. Thermal processing (even for short periods) can decrease the total phenolic content and reduce the antioxidant activity of plant materials [35,38]. This could explain the differences that different studies have reported for the same residues. The following residues have been studied as possible sources of ADF.

### 4.1. Avocado

Avocado byproducts (remains of pulp, peel, seed, and leaves) have been considered as sources of bioactive compounds due to their polyphenol contents [17] (hydroxycinnamic acids, hydroxy-benzoic acids, flavonoids, and proanthocyanins), as well as their contents of acetogenins, phytosterols, carotenoids, and alkaloids [18]. Polyphenols are distributed in the pulp, peel, seed, and leaves, while carotenoids and tocopherols are mainly found in the avocado pulp [18]. The peel and seed are also sources of fermentable sugars and fiber [39]. Natural extracts of the seed are rich in phenols with antioxidant properties [2], with higher levels than those reported for the pulp and for common synthetic antioxidants such as Trolox [39]. Phytochemical studies on avocado seeds have identified compounds such as saponins, phytosterols, triterpenes, fatty acids, furanoic acids, flavonol dimers, and proanthocyanidins. Protocatechuic acid was the main phenolic compound found, followed by kaempferide and vanillic acid [21]. The seed extract possesses low toxicity [21]; however, some authors have reported that at concentrations of 500 mg/kg, the extracts display toxic and genotoxic activity in mice [17,40,41]. Studies with hypercholesterolemic mice have demonstrated the reduction of cholesterol and low-density lipoproteins by the seeds, an effect attributed to their phenolic content, antioxidant activity, and dietary and crude fiber content [21].

Avocado residue extracts have been reported to have numerous biological activities useful in the food and pharmaceutical industries. Therefore, they could be used as sources of fiber and phenolic compounds [18].

### 4.2. Mango

Mango is one of the most consumed fruits. The peel is the main byproduct of processing [42,43], constituting about 15–20% of the total weight of the fresh fruit [44]. There is much interest in the study of mango peel due to the large quantities generated by the concentrate industry and its potential for use as an alternative ingredient in different food matrices. It is considered a good source of bioactive components [45] such as dietary fiber; compounds with antioxidant activity; phytochemicals such as polyphenols, carotenoids, vitamin E, and vitamin C; and enzymes [43,44]. Mango peel is also a good source of pectin, cellulose, hemicellulose, lipids, proteins, and reducing and nonreducing sugars, which may vary according to variety [46]. Among the main phenolic compounds reported in mango peel are syringic acid, quercetin mangiferin pentoside, and ellagic acid [43]. Some studies have reported that the main phenolic compounds present in mango participate in synergistic or antagonistic interactions that modify antioxidant capacities. However, the connection between the structure of these bioactive compounds and their biological activity is still under investigation [8].

Mango peel is a good source of dietary fiber (soluble and insoluble) [43,47]. It can be used for the extraction of bioactive compounds. In addition, the residue obtained can be used in the preparation of foods rich in dietary fiber [42,46] or as a prebiotic [47].

### 4.3. Papaya

Papaya byproducts (peels, seeds, and pulp) contain large amounts of nutrients, including dietary fiber and phenolic compounds with antioxidant activity [22,48]. Multiple phenolic compounds have been identified in papaya byproducts (protocatechuic acid hexoside, mangalin, quercetin 3-O-rutinoside, caffeoyl hexoside, and ferulic acid), as have lutein, zeaxanthin, β-carotene and β-cryptoxanthin, carotenoids, and ascorbic acid [22]. In total, 65% of the polyphenols associated with these dietary fiber concentrates are highly bioaccessible in the small intestine, and the nondigestible fiber portion shows antioxidant capacity [22]. Papaya peel contains vitamins like vitamin A, vitamin C, riboflavin, thiamin, and niacin. It is a source of phenols, alkaloids, flavonoids, tannins, and saponins [49]. The physicochemical properties of papaya peel vary by geographic location, variety, and season, which may affect processing and other associated activities. Therefore, extensive research is required [49].

According to Calvache et al. [22] the phenolic compounds found in dietary fiber concentrates from papaya peel were twice those found in the pulp (0.99 vs. 0.47 g/100 g). On the other hand, it was found that about 22% of the polyphenols present in fresh papaya pulp and more than 37% of the polyphenols present in the peel remained in the fiber after the concentration process.

### 4.4. Pineapple

During pineapple processing, a series of residues are generated, including peels (30%), pomace (50%), stems, crowns (13%), and fruit cores (7%). These residues or byproducts represent between 25 and 35% of the total weight of the fruit [36]. They consist of structural carbohydrates, dietary fiber, simple sugars, vitamins, and polyphenols [50]. The carbohydrates present in pineapple peel are bound to other compounds such as soluble fiber and polyphenols [12,50]. The main polyphenols identified in pineapple peels are gallic acid (31.76 mg/100 g of dry extracts), catechin (58.51 mg/100 g), epicatechin (50.00 mg/100 g), and ferulic acid (19.50 mg/100 g) [51]. Because of this, pineapple byproducts are considered biomass that can be exploited as a source of dietary fiber [50].

### 4.5. Grape pomace

After the vinification process, more than 70% of the grape polyphenols remain in the pomace [52]. This waste from the wine industry is mainly made up of peel, residual pulp and stalks, and seeds. These polyphenols structurally have one or more aromatic rings and are usually found as esters, methyl esters, or glycosides, which can be conjugated with mono-, oligo-, or polysaccharides in plant tissues [6,53].

### 4.6. Carrot

Carrot pomace is composed mainly of an insoluble, fiber-rich fraction, in which the presence of peptic polysaccharides, hemicellulose, and cellulose stands out. Studies have identified significantly enhanced functional properties, such as glucose-absorption capacity and amylase-inhibition activity, compared to those of cellulose. As carrot pomace is available in large quantities as a byproduct of juice production, it could be exploited as a good source of dietary fiber [54]. However, it has been reported that at drying temperatures above 90 °C, 20% of the β-carotene in carrots is degraded [35,55].

## 5. Action Mechanisms

**Figure 1:**
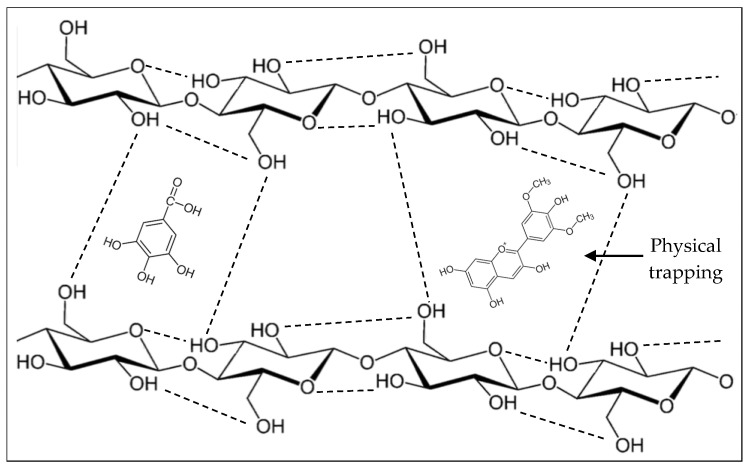
Physical trapping of phenolic compounds in cellulose fibers.

**Figure 2:**
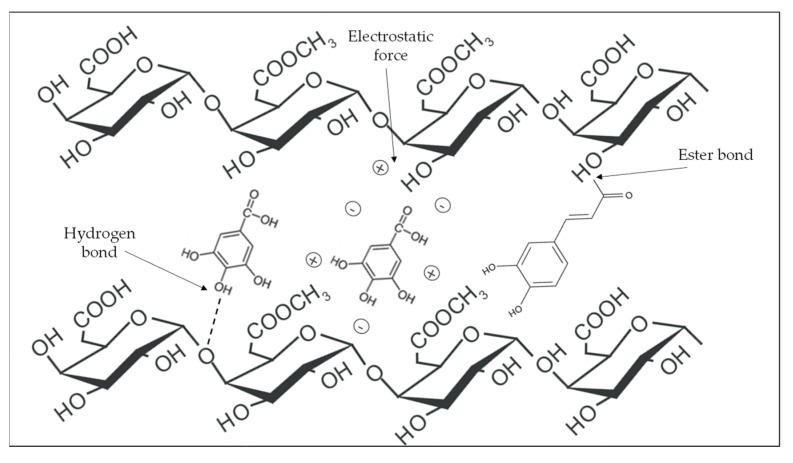
Physicochemical interactions between phenolic compounds and dietary fiber (pectin).

Phenolic compounds may be trapped within the complex of polysaccharide compounds that make up the fiber (Figure 1) or bound through chemical interactions. Binding through the hydrophobic aromatic rings and hydrophilic hydroxyl groups of phenolic compounds to polysaccharides and/or proteins takes place on the surface of the cell wall. [13,56]. Figure 2 shows the interactions between the hydroxyl groups of phenolic compounds and the oxygen atoms of the glycosidic bonds of polysaccharides, as well as the covalent bonds (ester bond) between phenolic acids and polysaccharides [11,13,57].

A large percentage of polyphenols are associated with dietary fiber. According to the type of bonding and the nature of the phenolic compound, these polyphenols can be considered extractable and nonextractable (attached to the cell wall). During food digestion (gastric or small-intestine phase), some compounds are released. Those of higher molecular weight, such as tannins and proanthocyanidins, covalently bound to dietary fiber or proteins, can only be released under more drastic conditions such as hydrolysis with sulfuric acid or enzymatic action [58]. The amount of nonextractable polyphenols is different in each food and depends on its nature; in foods such as wheat, barley, or coffee, it can be higher than 50% [58].

**Figure 3:**
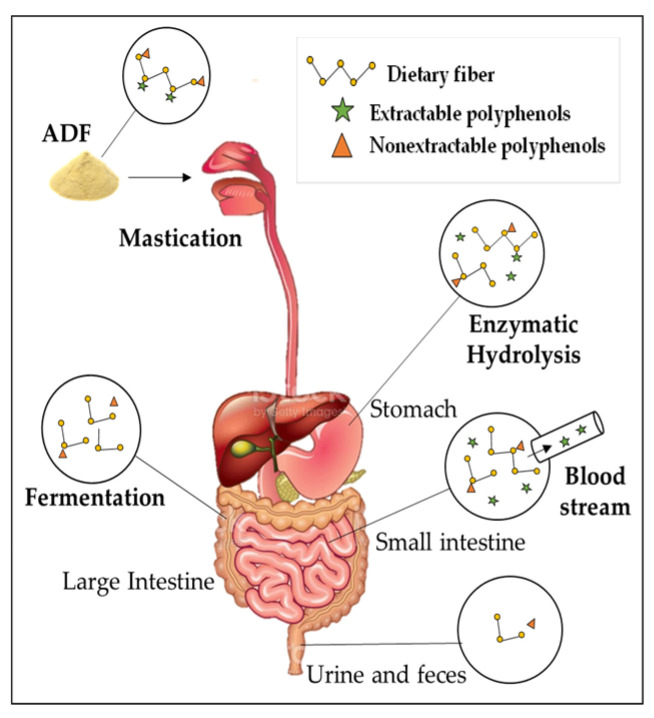
Bioavailability of phenolic compounds associated with dietary fiber.

As can be seen in Figure 3, some extractable polyphenols can be adsorbed in the small intestine, or reach the colon bound to dietary fiber, resistant protein, and starch. In the colon, ADF can be fermented by bacterial microflora, releasing metabolites that have been associated with human health benefits [59]. In some cases, these benefits have been related to the antioxidant environment generated by the phenolic compounds released after fermentation [50].

The use of byproducts as sources of ADF provides an opportunity to add value to primary production and mitigate the negative environmental impacts of poor disposal [35,39]. ADF obtained from these byproducts can be considered a functional ingredient because it improves the nutritional quality of food; can increase water-holding capacity, oil-holding capacity, emulsification, and/or gel formation; and improve texture, sensory characteristics, shelf life, and antioxidant activity [1,10,23].

## 6. Benefits of Fiber Consumption

There are conflicting results regarding the action of antioxidants in reducing the risk of developing some chronic diseases. Intervention studies have not fully confirmed the beneficial effects. Different factors can affect the action of phenolic compounds, including dosage, interaction with the food matrix, and bioavailability of polyphenols. Along with endogenous factors including the gut microbiota and digestive enzymes, the food matrix can also significantly affect the bioaccessibility, absorption, and subsequent metabolism of polyphenols [58]. At the same time, bioavailability may be conditioned by molecular interactions between the bioactive compounds and the food matrix [8].

Both dietary fiber and polyphenols have been linked separately to different health benefits; however, recent research suggests that these two important health-promoting components act in tandem [8,60]. Some studies have associated a greater intake of dietary fiber with a decreased risk of coronary heart disease, diabetes, obesity, and some forms of cancer [1], and with bile-acid retention–excretion, gastrointestinal laxative, hypoglycemic, hypocholesterolemic, prebiotic, and cardioprotective properties.

The functionality of fiber is associated with a reduced risk of cardiovascular diseases, diabetes, obesity, certain types of cancer, and intestinal diseases, all of which are directly related to the different physiological functions of fiber and its physicochemical properties, such as water-holding capacity (WHC), oil-holding capacity (OHC), swelling capacity (WSC), glucose-absorption capacity (GAC), and cholesterol-absorption capacity. (CAC) [23]. The physiological and metabolic effects of DF are related to the physicochemical, functional, and nutritional properties of the product. For example, fiber with a high water-holding capacity can directly influence the volume and mass of intestinal contents; highly viscous water-soluble dietary fiber (WSSF) exhibits a glucose-diffusion-hindering effect and helps to postpone carbohydrate absorption and digestion, resulting in a decrease in postprandial blood glucose and an increase in bile-acid excretion, which also contributes to a decrease in serum plasma cholesterol [60].

DF has an important role as a prebiotic, which is why it has been considered in the development of new functional foods in recent years [61]. Other advantages offered by DF are improved emulsion stability, texture, cooking performance, water-retention capacity, and sensory properties when it is incorporated into meat product formulations, in addition to inhibiting lipid peroxidation and microbial growth, prolonging the shelf life of meat and meat products [62]. Many studies have focused on the valorization of byproducts via the extraction and utilization of phenolic compounds [4]. However, this valorization leaves behind a residue that in many cases can be considered ADF. These dietary fibers could be incorporated into food formulations; in this regard, researchers have focused more on food reformulation strategies than on sensory or consumption aspects [63]. There is a need to study the use of ADF as a food ingredient in different formulations [2]. Technical, sensory, consumption, economic, and sustainability aspects should be evaluated [63], as well as optimizing the dosage of the products designed in animal models and carrying out clinical trials in humans to determine the effect of these formulations [2,61].

### 6.1. Pancreatic-Lipase-Inhibitory Effect

The insoluble dietary fiber present in citrus peels has an inhibitory and absorptive effect on pancreatic lipase activity. This ability is due to the structural and conformational changes of pancreatic lipase generated by its binding with the insoluble dietary fiber, which occur through a dynamic extinction process induced by electrostatic interactions with a single binding site between them. This union produces an increase in the hydrophobicity and a reduction in the polarity of the tyrosine (Tyr) and tryptophan (Try) residues, which explains the conformational alterations [64].

### 6.2. Prevention of Obesity and Cardiometabolic Diseases

The consumption of dietary fiber in the diet favors energy homeostasis. The presence of dietary fiber in food increases its density and decreases caloric intake [65,66]. The feeling of satiety generated after the consumption of foods rich in fiber reduces food ingestion. With the reduction of food intake (in some cases), a regulation of body weight is observed [67]. Soluble dietary fiber influences metabolic processes such as gastric emptying, starch hydrolysis, and diffusion of substrates and nutrients to the absorption surface [65]. These effects collectively lead to sustained glucose release (i.e., they reduce the glycemic index, which may help prevent type 2 diabetes (T2D) and obesity) [65]. However, the evidence is very mixed; there is no consensus on methodology and research is limited for many fibers. In general, viscous fibers show better results in appetite indices compared to nonviscous fibers [68]. Everyone will have a different response to the same diet, due to interindividual differences in genetic, epigenetic, microbiotic, and metabolic phenotypes. However, little research has been done to study the variation in response to dietary interactions based on the metabolic characteristics of individuals following dietary fiber intervention [69].

The consumption of dietary fiber has been associated for many years with a decreased risk of cardiovascular disease. Research on individuals has found that diets high in total dietary fiber (25 g/day) are associated with a lower risk of coronary heart disease and cardiovascular disease [70]. Kromhout et al. [71] conducted a 10-year study of risk indicators for coronary heart disease in which 871 middle-aged men from the city of Zutphen (the Netherlands) participated. During the evaluation period, a reduction in mortality from coronary heart disease and cancer associated with an increase in dietary fiber intake was demonstrated. Since then, much research has shown decreases in coronary heart disease risk associated with an increased dietary fiber in the diet [66]. Blood cholesterol is an important risk factor in coronary artery disease. A reduction in total cholesterol and low-density lipoprotein (LDL) decreases the risk of coronary heart disease.

One strategy for the prevention of cardiovascular disease is the reduction of cholesterol levels through the diet, using foods that can absorb cholesterol and bile acids or inhibitors that target their biosynthetic pathways [65]. Dietary fiber intake has been recommended as a safe alternative for cholesterol reduction [72] through multiple mechanisms, for example, the trapping of dietary cholesterol in the matrix formed by soluble fiber (lower impact), or the modification of cholesterol metabolism through short-chain fatty acids produced during fiber fermentation by intestinal microbiota, causing a reduction in the level of LDL cholesterol [70]. However, a study by Brown et al. [72] found that the cholesterol-lowering effect of dietary fiber was low within the practical range of intake. In this case, ingesting 3 g of oat soluble fiber (3 servings of 28 g) reduced total and LDL cholesterol by <0.13 mmol/L. The effects of fiber on cholesterol reduction may vary depending on the nature of the fiber. There are wide differences in the degree of cholesterol reduction caused by soluble fibers [72]. An example is oat products, for which results can vary between 18% and 0%, while pectin has produced results between 16% and 5%, and guar gum between 17% and 4% [72,73]. These marked differences are influenced by several factors, among which are the amount of fiber ingested, sample size, baseline diet, and changes in body weight, as well as the study population [72].

### 6.3. Effect of Fiber on the Intestinal Microbiota

For a food to be considered a prebiotic, it must meet three conditions: it must be resistant to gastric acid, mammalian enzymatic hydrolysis, and gastrointestinal absorption; it must have the ability to be metabolized by the intestinal microbiota; and it must stimulate the selective growth or activity of bacteria with beneficial health effects [74,75].

Some fibers can be classified as prebiotics if they are metabolized by beneficial bacteria present in the gut microbiota [74]. Dietary fibers provide metabolic fuel for the growth and/or proliferation of health-promoting bacteria that colonize the gastrointestinal tract [65]. Resistant oligosaccharides (fructans (fructooligosaccharides, oligofructose, and inulin) and galactans) have been widely documented as prebiotics, while other sources are considered to have prebiotic potential or to be prebiotic candidates and others do not present a prebiotic effect in humans [74]. As a result of the fermentation of dietary fiber by the gut microbiota, different end-products such as vitamins and short-chain fatty acids (SCFAs), including butyrate and propionate, are generated, which have healthy and beneficial effects.

A diet that provides fructooligosaccharides increases the percentage of beneficial intestinal microbiota, such as *Lactobacillus* and *Bifidobacterium* species. Fructans not only decrease gastrointestinal symptoms, but also increase gut immune function, reduce intestinal inflammation, and beneficially modulate the gastrointestinal microbiota [65]. A better understanding of the intimate interaction between dietary fiber and the intestinal microbiota may help in the development of new therapeutic strategies to prevent and treat diseases.

## 7. Antioxidant Dietary Fiber as a Food Additive and Natural Preservative

Different research reports have considered agroindustrial residues as natural and economical sources of dietary fiber (soluble and insoluble), essential amino acids, and phenolic compounds. The consumption of ADFs obtained from these byproducts has been associated with health benefits, and their use as functional ingredients in foods at low concentrations (0.5–3.0%) does not affect the physicochemical characteristics of foods. The development of food formulations incorporating these new functional ingredients is an emerging field. The latest products must meet a series of requirements for acceptability and functional characteristics demanded by today’s consumers [3,76]. Incorporating these byproducts into food formulations could help to mitigate the environmental impact caused by these wastes and improve the products’ nutritional quality.

One of the objectives of adding fiber to food formulations is to nutritionally enrich the products, increasing their fiber content as well as improving structural and textural properties [77]. Dietary fiber has been incorporated into baked goods, dairy products, jams, meats, and soups, with favorable results in texture, stabilization of fats and emulsions, improvement of shelf life, and, in some foods, prevention of syneresis [1]. Antioxidant dietary fiber provides not only the benefits of fiber, but also the benefits of the phenolic compounds present. Some foods even retain their antioxidant activity after heat treatment.

Table 3 presents a series of studies in which an agroindustrial byproduct (potential ADF source) was incorporated into a food formulation and its contribution to nutritional quality, functional properties, and/or acceptability was evaluated.

Generally, for the preservation of meat products, “sulfites” are used (a term that refers to sulfur dioxide and different forms of sulfur agents). Sodium or potassium bisulfite, as an antioxidant agent, is responsible for inhibiting microbial growth, as well as delaying discoloration. These agents are effective against Gram-negative aerobic bacteria, molds, and yeasts [85]. Their use has been questioned due to potential negative effects on health, such as asthmatic reactions and, in high concentrations, deficiency of thiamin or vitamin B1. For this reason, their use is limited in some countries. The estimated safe daily intake is 0–0.7 mg/kg per person per day, an intake that is exceeded in some cases due to the consumption of meat products. According to a FAO/WHO report on food additives (Safety evaluation of food additives, 2009), the investigation of alternative conservation methods is recommended to reduce the concentrations of SO_2_.

Other antioxidants used to control lipid oxidation in meat, such as butylated hydroxytoluene (BHT), butylated hydroxyanisole (BHA), propyl gallate (GP), and tertbutylhydroquinone (TBHQ), among others, have also raised concerns about their possible harmful effects [86], which has created a need for and fueled research on alternative antioxidants, particularly from natural sources [87].

The use of byproducts with antioxidant and antimicrobial properties as natural preservatives to prolong shelf life in foods is a promising technology [22,88]. Some research has shown the possibility of reducing the amount of sulfites in the formulation of cooked meat products, with the possibility of extending shelf life, by adding natural compounds with antioxidant activity such as antioxidant dietary fiber [89,90,91].

## 8. Conclusions

Byproducts of fruit and vegetable processing are important sources of antioxidant dietary fiber. Their low cost and the accessibility of large quantities make these byproducts viable raw materials for this use, in addition to the balance between the contents of soluble and insoluble fiber, which is directly related to a greater functionality of the fiber (compared to fiber from other sources such as cereals and mushrooms). Multiple studies have identified agroindustrial byproducts as sources of ADF. Research on the contents of bioactive compounds present in ADF, ADF’s function (in vivo), and its multiple applications in the food and pharmaceutical industry is currently ongoing. However, more research is needed regarding the interaction with the food matrix, bioavailability during processing and storage, and other compounds present that may affect safety, which should be studied before its possible commercial application.

Among the most outstanding uses of ADF is its application as a functional ingredient to enrich foods, due to its contribution of dietary fiber, antioxidants, antimicrobials, colorants, flavorings, and thickeners. In the same way, it can be used as a partial or total replacement for preservatives in processed foods, especially meat products.

## Figures and Tables

**Table 1:** Total dietary fiber (TDF) and total phenol content (TPC) of agroindustrial byproducts considered sources of antioxidant dietary fiber.

Source	Total Dietary Fiber (g/100 g)	Total Phenol Content (TPC) (mg GAE/g) ^1^	Reference
**Avocado**	**Pulp** (1.4–3); (4.10) **Leaf** **Peel** (1.29–54.63) **Seed** (2.19–4.24)	**Pulp** (0.61 to 16.81); (0.94–32.67); (4.10) **Leaf** (17 to 43.82) **Peel** (1.81 to 227.90); (1.58–172.18); (4.3–172.2); (6.79) **Seed** (1.55 to 292); (0.94–924.64); (5.7–88.2); (292); (7.04)	[17,18,19,20,21]
**Mango**	**DFC** ^2^ (70)	**DFC** (546) *; (283) **	[16]
**Papaya**	**Pulp** DFC (59.8) **Peel** DFC (53.8)	**Pulp** DFC (0.47) **Peel** DFC (0.99)	[22]
**Pineapple**	**DFC** (75.8); (51)	**DFC** (129); (1.49); (9.1); (2.6–51.1)	[16,23,24,25]
**Guava**	**DFC** (69.1); (43.21)	**DFC** (39) **; (2.43); (44.04) **Peel** (77.9) **Pulp** (26.2)	[16,26,27,28]
**Orange**	**Peel** (71.62); (69)	**Peel** (40.67); (9.61–31.62) **Leaf** (12.54–44.41) **NOP-IDF** ^3^ (1.47–6.982) **BP** (0.84–6.98)	[29,30,31]
**Passionfruit**	**Seed** DFC (81.5); (85.9)	41.2	[10,16,24]

^1^ GAE: gallic acid equivalents. ^2^ DFC: dietary fiber concentrate (mango coproducts were mainly peel and pulp; pineapple coproducts were mainly peel and heart; guava and passionfruit coproducts were mainly peel, pulp, and seeds). ^3^ NOP-IDF: insoluble dietary fiber of navel orange peel. BP: bound polyphenols. * methanol: acetone extractions; ** ethanol extraction.

**Table 2:** Antioxidant activities of fruit pulp, byproducts, and dietary fiber concentrates (DFCs).

Source	ABTS (µmol TE/g)	FRAP (µmol TE/g)	DPPH (µmol TE/g)	Reference
**Avocado**	**Peel** (112–791.5) **Seed** (91–725); (173.3)	**Peel** (23100) **Seed** (9500)	**Peel** (38–310) **Seed** (128.3–410.7)	[17,21]
**Papaya**		**Pulp** (10.2) **Peel** (25)	**Pulp** (12) **Peel** (54.86)	[22]
**Guava**	**(4.7)**	(10.96) **Peel** (392) **Pulp** (233)		[27,28]
**Orange**	**BP** (960–4100)		**BP** (12.96–30.97)	[31]

BP: bound polyphenols.

**Table 3:** Studies of incorporation of agroindustrial byproducts as sources of fiber and antioxidants.

Byproduct	Product	Added As	Effect	Reference
Pineapple pomace powder (PPD)	Yogurt	✓ Pomace was added (0%, 0.1%, 0.25%, and 0.5%)	✓ Increased dietary fiber ✓ Concentrations of 0, 0.1, and 0.25% showed good acceptability	[23]
	Vienna-type sausages	Chemical, physical, and technological properties were determined to select one pineapple and to evaluate the effect of its mixture with meats on characteristics of Vienna-type sausages	✓ Increased antioxidant carotenoids and polyphenols	[77]
	Donuts, meat patties, and golden layer cakes	Functional ingredient for bakery and meat products	✓ Higher dietary fiber content ✓ Improved physicochemical properties of products	[78]
	Cookies Particle sizes 400–251 µm, 250–150 µm, and ≤149 µm) and concentrations 5, 10, and 15% into refined wheat flour	Nutritional and functional properties of PPD were evaluated and the effect of PPD incorporation on dough and cookie quality was determined	✓ Increased content of dietary fiber (1.79–2.45%) and carbohydrates. ✓ Decreased protein and fat content. ✓ There were no differences in the physicochemical characteristics ✓ Low-gluten cookies with antioxidant activity	[79]
Cashew apple residue	Low-fat hamburgers	✓ 0 %–14.27 % cashew apple residue	✓ Improved yield ✓ Higher dietary fiber content (0 to 7.66%) (higher insoluble fiber content) ✓ Reduced lipids (35%) ✓ Decreased moisture and protein ✓ Increased carbohydrates and pH	[80]
Avocado peel extract (APE)	Beef and soy burgers	✓ 0.5 % and 1 % APE	✓ Decreased concentrations of TBARS, hexanal, and carbonyls (days 1 and 10) after cooking. ✓ Beef patties: addition of APE produced greater preservative effect than the control (sodium ascorbate) ✓ The addition of 0.5% APE inhibited the formation of heterocyclic aromatic amines and acrylamide in beef and soybean patties ✓ Modified color without affecting acceptability ✓ APE can be an alternative to synthetic antioxidant	[81]
Red grape pomace	Chicken Hamburger (raw and cooked)	✓ 0.5 %, 1 %, 1.5 %, and 2% grape antioxidant dietary fiber (GADF) ✓ Time: 0, 3, 5, and 10 days (4 °C)	✓ Improved oxidative stability and antioxidant activity ✓ The addition of GADF did not affect overall acceptability ✓ GADF was a very effective inhibitor of lipid oxidation and has potential as a natural antioxidant in raw and cooked chicken meat	[82]
Grape antioxidant dietary fiber (GADF)	Minced fish muscle (MFM)	✓ 0 %, 2 %, and 4 % GADF was added to MFM samples ✓ Stored at −20 °C (6 months)	✓ Reduced lipid oxidation (3 months), stored frozen	[83]
Wine grape pomace. ✓ Pinot Grigio WGP (WWGP) ✓ Pinot Noir WGP (RWGP)	Breads, muffins, and brownies	✓ 5%, 10%, 15% for bread (RWGP) ✓ 10%, 15%, 20%, 25% for brownies (WWGP) ✓ 5%, 10%, 15% for muffins (RWGP) ✓ 10%, 15%, 20% for muffins (WWGP)	✓ This study reported that a 5.9% or 194.4% increase in polyphenols and a >20% dietary fiber increase could be achieved in pomace-fortified breads or muffins, respectively, without impacting consumer acceptance of the products	[6]
Mango peel	Beef burger	✓ 3 %, 6 %, 9 %, 12 % mango peel dietary fiber	✓ Increased contents of dietary fiber, polyphenols, and carotenoids in hamburger meat ✓ Improved antioxidant activity	[84]

## Data Availability

Data are contained within the article.
